# Quality of Life in Kidney Donors: A Single-Center Experience

**DOI:** 10.7759/cureus.52481

**Published:** 2024-01-18

**Authors:** Lamees Alayoobi, Hend A Aljenaidi, Wejdan Alqassab, Waleed Kaabi, Hasheem Alzeyedi, Abdullah Asiri, Othman Alhumaidan, Nadyah Aeknaifeth, Ahmed Jaradat, Amgad E El-Agroudy

**Affiliations:** 1 Nephrology, Salmaniya Medical Complex, Manama, BHR; 2 Internal Medicine, Arabian Gulf University, Manama, BHR; 3 Family and Community Medicine, Arabian Gulf University, Manama, BHR; 4 Nephrology, Urology & Nephrology Center, Mansoura University, Mansoura, EGY

**Keywords:** single-center study, bahrain, sf-36 questionnaire, kidney donors, quality of life (qol)

## Abstract

Background: Live kidney donation is used to treat end-stage renal disease, but it poses risks and decision-making challenges for donors. This study examines kidney donors' long-term quality of life (QOL).

Methods: We conducted a cross-sectional survey on kidney donors between 1982 and 2018. We used the Short Form-36 (SF-36) (Arabic version) to measure the donors' QOL. Out of 60 donors contacted, 44 agreed to participate and responded. Demographic information, donor-recipient relationships, and specific questions about the donation were collected and analyzed.

Results: The mean age of the donors was 50.1 ± 11.7 years at follow-up, and 26 (59.1%) were males. Most donors were siblings and parents, accounting for 36.4% (n=16) each. The time since the donation was 111.5 ± 97.1 months. All donors decided voluntarily to donate and all of them would do it again if given a chance. However, one donor (2.3%) complained that the donation had caused problems in his marriage, while six donors (13.4%) experienced clinically relevant distress, and two donors (4.5%) experienced financial disadvantages. Donors had high QOL scores, with a mean score of 73.1 and 96.9 (on a scale of 1-100) for the eight subscales. The highest score was for role social functioning, while the lowest was for energy/fatigue. The mean scores for the four fatigue subscales were low, ranging from 61.8 to 86.8. The lowest score was for feeling calm and reassured, while the highest was for feeling frustrated. In social functioning, the highest score was for the effect of mental health on work performance (97.2). We analyzed various demographic factors and their correlation with QOL and found no significant correlation in most domains regarding sociodemographic characteristics.

Conclusions: Our research indicates that most kidney donors have had a positive experience over the past four decades. Both male and female donors of all ages reported good long-term QOL, further enhanced by recognition and support from their families and friends. These findings provide further support for our current policy on organ donation.

## Introduction

Kidney transplantation is considered the most effective treatment for end-stage renal disease (ESRD). Unfortunately, many transplant centers face a shortage of kidney donors. This is because some people are afraid of the surgery and its potential complications, as well as lifestyle changes and post-operative limitations. Additionally, there is a fear of the remaining kidney function deteriorating or developing a kidney disease that would further affect their health [1]. On the other hand, each kidney donor has their unique motivation behind donation - whether to save a loved one's life or boost their self-esteem.

Studies have shown that donating a kidney does not lead to serious medical problems such as a decline in kidney function, arterial hypertension, or proteinuria [2,3]. However, the donor's quality of life (QOL) results are mixed. While some studies [4-14] suggest that organ donors have a QOL comparable to or better than the general population, others report a negative impact on their QOL [15-19]. Some studies have shown that donors experience positive outcomes such as enhanced self-worth. In particular, a report from one center showed that donor candidates displayed high resilience and QOL despite the stress of the donation [5]. In another report of 111 kidney donors, the majority of these donors had positive experiences; they added that they would donate again if given the choice because they experienced enhanced self-worth [7]. In Arabic countries, cultural and religious beliefs may encourage more people to donate organs [4]. A study from Saudi Arabia suggested that donors had a good QOL, which could motivate further donations [4]. However, some studies have also found that donors may be at a higher risk of developing psychiatric disorders. Up to 5% of donors may not donate again. Additionally, up to 15% of donors believe that donating negatively affects their health, and between 16% and 23% of donors report negative financial consequences [8,16]. A study from Iran found that most donors contributed due to financial hardship [20]. The RELIVE study, which examined kidney donors' long-term medical and psychosocial outcomes at three US transplant centers, reported mostly positive outcomes, with around 1 in 10 donors reporting at least one negative outcome [6].

There is generally a lack of long-term data regarding the problems experienced by the population who donate. This study is unique because it involves a group of people monitored for over 40 years. The study aims to examine the factors that affect long-term self-reported QOL, with a specific emphasis on various ethnic groups.

## Materials and methods

Patients

A study was conducted between 1982 and 2018 to gather information from kidney donors through a cross-sectional survey. Participants were contacted through postal mail, and the invitation letter explained the study's purpose while ensuring confidentiality. If no response was received within a month, another set of questionnaires was sent. If the patient still failed to respond, they were contacted either through their recipients or by phone. If a participant refused to participate, the reasons for their non-participation were recorded. Each study participant provided informed consent, and non-participation was considered their legal right. Initially, ethical approval was obtained from the Ethical Committee of the Institutional Review Board (IRB) at Arabian Gulf University (E32-PI-2-23) and Salmaniya Medical Complex (SMC), Governmental Hospitals, Bahrain (16070323).

Donor-specific questions

We obtained demographic information from the donors, including age, gender, education level, employment status, marital status, and their relationship with the recipients. In the questionnaire, we provided five possible responses: "Definitely," "Probably," "Don't know," "Probably not," and "Definitely not." In the second part of the survey, we asked about the donation process. These questions included how donors received information about donation, any stresses related to the donation process, any expenses incurred, any physical and emotional changes experienced during the process, as well as preexisting factors that may affect the donation process, such as marriage, level of education, relationship to the donor and motivation for donation. Their medical records gave us clinical information on postoperative or long-term medical complications.

Short form-36 questionnaire

We evaluated the overall QOL for organ donors by using SF-36, which is a trustworthy questionnaire tool available in the Arabic version [4]. This generic QOL tool consists of 36 questions and eight subscales that represent different aspects of health, such as physical functioning (10 items), role limitations due to physical health problems (four items), bodily pain (two items), general mental health (five items), energy and vitality (four items), social functioning (two items), role limitations due to emotional problems (three items), and mental health (five items). These subscales can be combined to form two components: the physical component score and the mental component score (MCS). To get the final score, all the questions must be answered, and each subscale is scored from 0 (least well-being) to 100 (greatest well-being).

Statistical analysis

We initially used an Excel spreadsheet to record and verify data, which we later exported to IBM SPSS Statistics for Windows, Version 24 (Released 2016; IBM Corp., Armonk, New York, United States). Descriptive analysis results were presented as mean ± SD. We used a one-way analysis of variance method and Student's t-test to examine the relationships between sociodemographic variables (such as gender, age at last follow-up and at the time of donation, employment status, level of education, relationship to recipients, and time after donation). We used Pearson's correlation coefficient to analyze QOL scores, evaluate the inter-domain correlation, and determine the correlation between demographic variables and domain scores. We considered P-values of <.05 to be statistically significant.

## Results

Donors

Out of 60 donors, 44 responded to the questionnaires sent by the Department of Nephrology at Salmaniya Medical Complex, Governmental Hospitals, Bahrain, which means a response rate of 72.3%. Unfortunately, out of the 16 donors who didn't reply, 15 had moved and could not be contacted, and one refused to participate. Of the 44 donors who participated, 26 were male (56.8%) and 18 were female (40.9%). The average age of the donors at the time of donation was 34.9 ± 9.7 years, while at the time of the study, it was 50.1 ± 11.7 years. The average duration between donation and participation in the study was 111.5 ± 97.1 months (with a median of 83 months). All the donors were related to the recipient, with siblings (n=16; 36.4%) and parents (n=16; 36.4%) being the most common. None of the donors felt compelled or had any doubts about donating. At the time of donation, 27 donors (61.4%) held jobs, while 9 (20.5%) were unemployed. All the donors were educated, with the majority (n=21; 47.7%) having a high school diploma and only three donors (6.8%) having postgraduate degrees (Table 1).

**Table 1:** Demographic Characteristics of the Participants in the Study Data represented as N and %.

		n	Percent
Age in years	< 30	4	9.1
	>= 30	40	90.9
Age at donation	< 30	19	43.2
	>= 30	25	56.8
Gender	Male	26	59.1
	Female	18	40.9
Duration after donation	< 5	15	34.1
	>= 5	29	65.9
Educational level	Less than High School Diploma	5	11.4
	High School Diploma	21	47.7
	University Graduate	15	34.1
	Postgraduate Studies	3	6.8
Current chronic diseases	No	34	77.3
	Dm	2	4.5
	Hypertension	8	18.2
Relation to recipient	Parents	16	36.4
	Siblings	16	36.4
	Offspring	7	15.9
	Other Relative	5	11.4
Social relationship after donation	Improved after Donation	21	47.7
	Same before Donation	22	50.0
	No Communication	1	2.3
Social relationship after donation	Improved After Donation	21	47.7
	Same before Donation	22	50.0
	No Communication	1	2.3
Work before donation	Office	18	40.9
	Manual	9	20.5
	Retired	8	18.2
	Unemployed	9	20.5
Effect of donation on work performance	Same before Donation	29	65.9
	Increase after Donation	1	2.3
	Decrease after Donation	7	15.9
	Not Working	7	15.9
Effect of donation on annual Income	No effect	38	86.4
	Affect Positively	4	9.1
	Affect Negatively	2	4.5
Donation effect on family relationships	No	43	97.7
	Yes	1	2.3
	Total	44	100.0

Medical data

All the donors who underwent surgery did not face any major complications such as pain, wound issues, or prolonged hospital stays. They were discharged after 5-6 days post-surgery. Currently, 34 donors (77.3%) do not have any medical problems. Among the remaining donors, only 2 (4.5%) have diabetes mellitus, while eight donors (18.2%) have hypertension, and most of these donors (n=27, 61.4%) were above 50 years of age.

Financial and occupational issues

During the survey, donors were asked if their donation affected their financial situation. Out of the donors who participated in the survey, 95.5% (42 donors) did not face any financial difficulties, whereas 4.5% (2 donors) reported experiencing financial disadvantages All of the donors retained their jobs after making the donation. However, out of the total employed donors, 15.9% (7 donors) faced temporary work interruption before and after the donation, which caused some challenges with their employers. On the other hand, 70.2% (30 donors) performed well at work after the donation.

Social issues

Forty-four donors (97.5%) perceived their relationship with their families to be closer, while only one donor (2.3%) complained that the donation disturbed his marriage.

QOL questionnaire

The participants in the study had a high QOL, with mean scores ranging from 73.1 to 96.9 for the eight subscales. The highest score was for role social functioning, while the lowest was for energy/fatigue. The mean scores for the four fatigue subscales were low, ranging from 61.8 to 86.8. The lowest score was for feeling calm and reassured, while the highest was for feeling frustrated. In social functioning, the highest score was for the effect of mental health on work performance (97.2). When it came to role limitation due to physical health, the highest score was that the donation does not affect either time spent working (90.91) or the restriction to a particular type of work (90.91). The same result (90.91) was seen regarding role limitations due to emotional problems, specifically mental health's effect on work productivity (Table 2).

**Table 2:** Descriptive Statistics for Each Domain of the QOL Among the Studied Patients Data represented as Mean ± SD. QOL: Quality of life

Domain of Quality of Life	Mean	SD
Physical Functioning	94.89	9.37
What is your health compared to last year?	85.23	27.66
Health condition restriction to strenuous activity?	93.18	17.36
Health condition restriction to Moderate activity?	96.59	12.75
Health condition restriction to holding Purchases from supermarkets?	94.32	16.05
Health condition restriction to climbing stairs for several floors?	97.73	10.54
Health condition restriction to climbing stairs for one floor?	96.59	12.75
Health condition restriction to bending for prayer?	95.45	14.54
Health condition restriction to walking 1.5 Km?	95.45	14.54
Health condition restriction to walking 0.5 Km?	96.59	12.75
Health condition restriction to walking 100 m?	97.73	10.54
Role Limitations Due to Physical Health	89.77	28.18
Health condition restriction to showering and wearing clothes?	88.64	32.10
does your physical health causing reduced time spent in work or other activities?	90.91	29.08
does your physical health causing reduced productivity at work or other activities?	88.64	32.10
does your physical health causing restriction to special type of work or other activities?	90.91	29.08
Role Limitations Due to Emotional Problems	88.64	30.45
does your physical health cause difficulty in performing work or other activities?	86.36	34.71
does your mental health causing reduced time spent in work or other activities?	88.64	32.10
does your mental health cause reduced productivity at work or other activities?	90.91	29.08
Energy/Fatigue	73.07	13.82
During the past four weeks, to what extent has physical pain interfered with your regular activities (whether inside or outside the home)?	64.55	26.54
How long did you feel calm and reassured?	61.82	25.81
How long have you felt frustrated and hopeless?	86.82	23.60
How long have you felt like a happy person?	79.09	18.78
Emotional Well-Being	78.45	12.33
How long have you felt full of vitality and activity?	72.73	27.65
How long have you been so nervous?	90.00	16.98
How long have you felt depressed?	71.36	27.75
How long have you had so much energy?	89.09	23.01
How long have you felt exhausted (used up)?	69.09	31.54
Social Functioning	96.88	7.68
does your mental health cause you not to perform work or other activities in usual care?	97.16	9.67
How long have you felt tired?	96.59	8.68
Pain	91.48	15.16
During the past four weeks, to what extent has your physical or mental health interfered with your usual social activities with your family, friends, neighbors, or other social events?	88.64	19.95
During the past four weeks, how severe have you been experiencing physical pain?	94.32	16.05
General Health	79.89	12.55
Donation effect on marriage?	84.09	22.93
During the past four weeks, how much time did your physical or mental health interfere with your social activities (such as visiting friends, relatives, etc.)?	73.86	27.47
Do you get easily sick?	84.66	17.23
Is your health condition similar to others you know?	67.61	23.86
Are you thinking of your health deteriorating?	89.20	15.62
Health Change	56.25	20.26

Various demographic factors and their correlation with QOL are analyzed and presented in Table 3.

**Table 3:** Comparison of Domain Scores With Demographic Characteristics Data represented as Mean ± SD. Differences with P <0.05 were considered statistically significant, and <0.001 were highly significant.

	Physical Functioning	Role Limitations Due to Physical Health	Role Limitations Due to Emotional Problems	Energy/Fatigue	Emotional Well-Being	Social Functioning	Pain	General Health
	Mean ± SD	Mean ± SD	Mean ± SD	Mean ± SD	Mean ± SD	Mean ± SD	Mean ± SD	Mean ± SD
Age at donation								
< 30	91.32 ± 12.00	86.84 ± 29.31	84.21 ± 34.01	75.26 ± 16.20	78.74 ± 13.20	96.71 ± 7.02	83.55 ± 19.12	79.21 ± 13.97
>= 30	97.60 ± 5.61	92.00 ± 27.69	92.00 ± 27.69	71.40 ± 11.77	78.24 ± 11.89	97.00 ± 8.29	97.50 ± 7.07	80.40 ± 11.63
P-Value	0.023	0.277	0.204	0.182	0.448	0.452	0.003	0.380
Gender								
Male	94.81 ± 8.89	87.50 ± 31.02	85.90 ± 34.22	73.46 ± 13.25	78.62 ± 12.75	96.63 ± 9.05	91.06 ± 15.70	79.42 ± 14.38
Female	95.00 ± 10.29	93.06 ± 23.96	92.59 ± 24.40	72.50 ± 14.98	78.22 ± 12.06	97.22 ± 5.35	92.08 ± 14.76	80.56 ± 9.68
P-Value	0.474	0.263	0.240	0.412	0.459	0.403	0.414	0.386
Duration after Donation								
< 5	95.67 ± 10.33	93.33 ± 25.82	93.33 ± 25.82	73.67 ± 14.33	80.00 ± 11.51	99.17 ± 3.23	93.67 ± 13.43	82.00 ± 9.60
>= 5	94.48 ± 9.00	87.93 ± 29.60	86.21 ± 32.75	72.76 ± 13.80	77.66 ± 12.85	95.69 ± 9.01	90.34 ± 16.09	78.79 ± 13.87
P-Value	0.348	0.276	0.234	0.420	0.278	0.035	0.249	0.214
Educational level								
Less than a high school diploma	96.00 ± 8.94	100 ± 0.00	100 ± 0.00	67.00 ± 19.24	72.80 ± 13.68	95.00 ± 6.85	98.00 ± 4.47	81.00 ± 10.25
High school diploma	95.71 ± 6.94	91.67 ± 26.61	90.48 ± 30.08	73.57 ± 14.50	78.10 ± 12.11	97.02 ± 6.74	91.90 ± 14.94	79.76 ± 13.37
University graduate	93.61 ± 11.98	84.72 ± 33.36	83.33 ± 34.77	74.17 ± 11.66	80.44 ± 12.42	97.22 ± 9.15	89.17 ± 17.19	79.72 ± 12.77
P-Value	0.761	0.525	0.528	0.586	0.474	0.849	0.517	0.979
Current Chronic disease								
No	94.85 ± 9.00	89.71 ± 27.60	88.24 ± 30.58	73.09 ± 14.57	77.76 ± 13.33	96.69 ± 8.32	91.10 ± 15.65	80.15 ± 12.52
Yes	95.00 ± 11.06	90.00 ± 31.62	90.00 ± 31.62	73.00 ± 11.60	80.80 ± 8.18	97.50 ± 5.27	92.75 ± 14.07	79.00 ± 13.29
P-Value	0.483	0.489	0.437	0.493	0.250	0.387	0.383	0.401
Donor relation to recipients								
Parents	92.50 ± 12.91	87.50 ± 30.28	85.42 ± 34.36	72.81 ± 16.33	75.75 ± 12.00	96.88 ± 7.22	84.22 ± 20.55	79.38 ± 14.36
Siblings	96.25 ± 6.71	87.50 ± 34.16	87.50 ± 34.16	73.44 ± 14.11	78.00 ± 13.15	96.09 ± 9.92	94.84 ± 9.42	80.94 ± 13.07
offspring	95.00 ± 7.64	100 ± 0.00	100 ± 0.00	74.29 ± 8.38	82.29 ± 9.20	96.43 ± 6.10	98.57 ± 3.78	78.57 ± 6.90
other relatives	98.00 ± 4.47	90.00 ± 22.36	86.67 ± 29.81	71.00 ± 13.87	83.20 ± 15.34	100 ± 0.00	94.00 ± 13.42	80.00 ± 14.14
P-Value	0.602	0.782	0.767	0.982	0.545	0.809	0.101	0.977

Age was found to have a significant association with QOL in some domains. At the time of donation, older donors scored higher on the physical functioning scale (P= 0.023) and pain (P= 0.003) but not in other domains. According to the data, female donors had slightly higher scores in the physical Functioning domain, but this difference was not statistically significant (P < 0.05). On the other hand, female donors scored higher than male donors in role limitation due to physical health, role limitations due to emotional problems, social functioning, pain, and general health domains. The duration after donation only significantly affected the social functioning score. Donors who were over five years after donation had lower scores (95.69) compared to donors who had donated less than five years ago (99.17) (P =0.035). There was no relationship found between QOL scores and level of education or the presence of current chronic medical diseases.

Pearson's correlation coefficient analysis of the QOL domains shows that most domains have a significant positive correlation (p-value <0.005), except for social functioning (SF). SF has a weak correlation with all domains, which is not significant, and it is positive for all domains except for role limitations due to emotional problems (RL/EP), where it is negative (p-value >0.05). Additionally, there is no significant correlation between SF and pain or general health (GH) and between physical functioning (PF) and energy/fatigue (E/F) and SF (r=0.259, p-value = 0.09); r=0.278, p-value = 0.68) respectively (Table 4).

**Table 4:** Pearson’s Correlation Coefficient Among the QOL Domain Scores PF: Physical Functioning, RL/PH: Role Limitations Due to Physical Health, RL/EP: Role Limitations Due to Emotional Problems, E/F: Energy/Fatigue, EW: Emotional Well-Being, SF: Social Functioning, GH: General Health, QOL: Quality of Life Data represented as the Pearson correlation coefficient. Differences with P <0.05 were considered statistically significant, and <0.001 were highly significant

	PF	RL/PH	RL/EP	E/F	EW	SF	Pain	GH
PF	1							
		RL/PH	r 0.447^**^	1						
p 0.002								
RL/EP	r 0.430^**^	r 0.968^**^	1					
	p 0.004	p < 0.001						
E/F	r 0.259	r 0.329^*^	r 0.324^*^	1				
	p 0.090	p 0.029	p 0.032					
EW	r 0.333^*^	r 0.342^*^	r 0.357^*^	r 0.555^**^	1			
	p 0.027	p 0.023	p 0.017	p < 0.001				
SF	r 0.278	r 0.017	r -0.031	r 0.133	r 0.267	1		
	p 0.068	p 0.914	p 0.841	p 0.388	p 0.080			
Pain	r 0.648^**^	r 0.489^**^	r 0.453^**^	r 0.415^**^	r 0.306^*^	r 0.178	1	
	p < 0.001	p 0.001	p 0.002	p 0.005	p 0.043	p 0.248		
GH	r 0.306^*^	r 0.621^**^	r 0.605^**^	r 0.478^**^	r 0.549^**^	r 0.132	r 0.487^**^	1
	p 0.043	p < 0.001	p < 0.001	p 0.001	p < 0.001	p 0.394	p 0.001	
**. Correlation is significant at the 0.01 level (2-tailed).								
*. Correlation is significant at the 0.05 level (2-tailed).								

## Discussion

When analyzing the literature on living donors, retrospective studies may have recall bias, ranging from 5 to 20%. This is particularly true when collecting questionnaire data over 30 years after the surgery. In contrast, prospective studies are more likely to report negative outcomes, but these assessments are generally conducted only once shortly after the surgery, and it is unclear how long these effects persist. Another issue is selecting suitable control groups for comparison [17]. Living kidney donors are healthy individuals selected on that basis, making it difficult to find a suitable control group. Comparing living donors to national data from population surveys may underestimate the psychosocial morbidity associated with living donation, as questionnaire scores may be lower in a population survey due to the inclusion of individuals affected by acute and chronic illness. Our study is noteworthy because we received responses from over 73% of the donors who answered the questionnaire about their donations and daily lives. This response rate is higher than other studies, which noted only 50-60% response rates [5-7]. All the donors decided to donate easily, spontaneously, and voluntarily, as per their declaration in the questionnaire. We also obtained long-term information about the current health status of still-alive donors. The donors responded promptly with most replies received within three months. Our study found that the living donors were stable, and their scores were relatively high on all eight scales. The donors in our study were mainly parents and siblings, with a median age of 35 years at the time of donation. The duration range of donations was 40.3 years, with a median of 83 months.

According to our study, donors who had donated 40 years ago had an average general QOL score of 13.4 and reported clinically relevant distress. Specifically, they experienced decreased levels of energy/fatigue and emotional well-being. This included feeling exhausted, experiencing depression, and having problems with happiness or frustration at work or in other daily activities due to emotional issues. Our findings are consistent with previous studies [4-12], which suggest that donors are not at risk of developing clinically relevant distress in the first few years, post-donation, or even after a long duration. However, the risk may increase marginally after 40 years post-donation. Please note the following information: The time-related data from our sample should be interpreted cautiously due to the relatively small number of donors (18 donors) spanning over 10 years. In agreement with other studies, the QOL in our sample is generally comparable to their results [4-14]. Some other prospective studies have shown a decrease in SF-36 scores after donation, suggesting a negative impact. When conducting SF-36 questionnaires immediately post-operative, there is a decline in QOL scores, initially during the postoperative period due to the consequences of surgery [4,12]. However, SF-36 scores have been shown to return to preoperative levels later. Meyer et al. in their 10 years of study after donation, reported both excellent health values in several domains of QOL scores [14]. Our study shows that good health appears to be sustained long-term. Thus, our results provide good knowledge about the long-term health outcomes that may impact the information provided to donors in the future.

The work confirms that donors perceive living-related kidney donation as safe and satisfactory. None of the donors experienced any doubts or pressure regarding the procedure. Many donors expressed their willingness to donate again, a common observation in various studies [4,5,9-14]. It is crucial to note that donating under any external pressure is considered a high-risk motive, as it may negatively impact the psychosocial outcomes of donors [21]. Previous studies [8,15,16] have indicated that donors who experience medical problems after donation are at an increased risk. We also found that donors who faced medical issues after donation were more likely to doubt the procedure. However, Zheng et al. found no such association [11].

In general, the number of donors who experienced emotional problems that affected their physical performance, such as difficulty with work or daily activities, was low in our sample. This finding is consistent with most studies [4,6,9-12]. Additionally, our study found that limitations in performing physical activities, such as climbing stairs or shopping, were also infrequent among donors. According to our research, 4.5% of donors experienced economic losses due to the donation process. Additionally, around 15.9% reported that their work performance was negatively affected. In a study by [22], 16.5% of donors incurred personal expenses, with 10.7% considering it an economic burden. Nevertheless, donors were not pressured to return to work before full recovery.

It is important to consider the cultural aspect of kidney transplantation in Bahrain, where living donors are typically limited to family members. According to our study, 97.5% of donors felt their relationship with their families improved after donating a kidney. Donors often experience psychological satisfaction in knowing that they saved the life of a family member by donating their kidney. Donors are typically optimistic, highly motivated, and altruistic individuals. A study conducted by Clemens et al. found that donors were more content, cheerful, and less likely to feel that life was meaningless compared to non-donors [23]. These positive emotional experiences can improve donors' and recipients' QOL and reduce anxiety and depression. In contrast, in Iran, 90% of donated kidneys were from non-related donors who primarily intended to receive financial incentives provided by the government [20]. A study conducted by Nejatisafa et al. showed that the QOL of these donors was inferior to that of the control group, and this may experience enduring pressures over an extended period, which may arise from familial responsibilities, work-related stress, or economic challenges [20].

In this study, the impact of age on organ donation was examined. In the long run, we observed no significant difference in reported QOL scores between younger and older donors. This finding is consistent with previous research [12,24] that looked at short-term results and is supported by the results of the long-term RELIVE study [6]. For example, in a study by Minnee et al., where donors more than 55 years old at the time of donation were considered elderly, older living kidney donors initially took longer to recover physically than younger donors [25]. However, after one year, the two groups had no significant differences.

The present study detected no significant distinction between males and females in all aspects of the SF-36 questionnaire. However, a retrospective investigation conducted by Sommerer et al. revealed a gender difference in QOL and fatigue, with a median of 77 months of follow-up [26]. Research on exhaustion in the general population has presented discrepancies in gender differences, which make it challenging to comprehend the pronounced fatigue observed in female donors in many studies [9,14,27].

Our findings revealed that only 18.2% of kidney donors had hypertension or received treatment. Furthermore, most of these donors (62.5%) were over 50 years. We found no significant differences when we compared the age-adjusted prevalence of hypertension among donors to the general population. El-Agroudy et al. reported a higher rate of hypertension (22.1%) among donors with an interval of more than 25 years from donation [2]. However, they also found that live kidney donation had no long-term effects on kidney function. Another study found no significant change in renal function or increased blood pressure for older donors [3]. Our results suggest no associations between clinical and self-reported outcomes or between hypertension and donor-specific factors.

Limitations

It is necessary to acknowledge the limitations of the study. Firstly, cross-sectional follow-up studies may lack baseline data and a control group, resulting in biased results. Secondly, self-reports up to 40 years later may be affected by recall bias and may have influenced the accuracy of the donor's memory of the distant past. However, this bias is mentioned in many long-term cross-section studies in the literature. The third limitation of the study was that it had a limited sample size and was conducted retrospectively.

## Conclusions

Our research reveals that most individuals who donated a kidney had a positive experience over 40 years. This confirms that donating a kidney from a living donor is safe and satisfactory. Furthermore, the majority of donors expressed a willingness to donate again. We recommend that donors not face any financial or occupational disadvantages due to their donation. Both younger and older donors of both genders reported good long-term QOL, which improved with the support of family and friends. Donors should not worry about major negative changes in the long term, as kidney function appears stable, and hypertension does not seem to occur more frequently compared to previous population-based studies. Although our findings support our current policy for donation, we need more participants in future prospective studies to enhance our understanding of the factors influencing the QOL of living kidney donors.

## References

[1] Cecka J (2002). The UNOS Renal Transplant Registry. Clin Transplant.

[2] El-Agroudy AE, Sabry AA, Wafa EW (2007). Long-term follow-up of living kidney donors: a longitudinal study. BJU Int.

[3] El-Agroudy AE, Wafa EW, Sabry AA (2009). The health of elderly living kidney donors after donation. Ann Transplant.

[4] Alhussain BM, Alqubaisi AK, Omair A, O'hali WA, Abdullah KO, Altamimi AR (2019). Quality of life in living kidney donors: a single-center experience at the King Abdulaziz Medical City. Saudi J Kidney Dis Transpl.

[5] Feltrin A, Pegoraro R, Rago C (2008). Experience of donation and quality of life in living kidney and liver donors. Transpl Int.

[6] Jacobs CL, Gross CR, Messersmith EE (2015). Emotional and financial experiences of kidney donors over the past 50 years: the RELIVE Study. Clin J Am Soc Nephrol.

[7] Jaseanchiun W, Sirithanaphol W, Chotikawanich E, Chau-in S, Pacheerat K, Uttaravichien T (2012). Quality of life after donor nephrectomy for living donor kidney transplantation at Srinagarind Hospital. J Med Assoc Thai.

[8] Isotani S, Fujisawa M, Ichikawa Y (2002). Quality of life of living kidney donors: the short-form 36-item health questionnaire survey. Urology.

[9] Fehrman-Ekholm I, Brink B, Ericsson C, Elinder CG, Dunér F, Lundgren G (2000). Kidney donors don't regret: follow-up of 370 donors in Stockholm since 1964. Transplantation.

[10] Lima DX, Petroianu A, Hauter HL (2006). Quality of life and surgical complications of kidney donors in the late post-operative period in Brazil. Nephrol Dial Transplant.

[11] Zheng XY, Han S, Wang LM, Zhu YH, Zeng L, Zhou MS (2014). Quality of life and psychology after living-related kidney transplantation from donors and recipients in China. Transplant Proc.

[12] Meyer K, Wahl AK, Bjørk IT, Wisløff T, Hartmann A, Andersen MH (2016). Long-term, self-reported health outcomes in kidney donors. BMC Nephrol.

[13] Glotzer OS, Singh TP, Gallichio MH, Conti DJ, Siparsky NF (2013). Long-term quality of life after living kidney donation. Transplant Proc.

[14] Meyer KB, Hartmann A, Mjøen G, Andersen MH (2017). Relationships between clinical, self-reported, and donation specific outcomes: a prospective follow-up study 10 years after kidney donation. Ann Transplant.

[15] Giessing M, Reuter S, Schönberger B (2004). Quality of life of living kidney donors in Germany: a survey with the Validated Short Form-36 and Giessen Subjective Complaints List-24 questionnaires. Transplantation.

[16] Smith GC, Trauer T, Kerr PG, Chadban SJ (2003). Prospective psychosocial monitoring of living kidney donors using the SF-36 health survey. Transplantation.

[17] Reimer J, Rensing A, Haasen C, Philipp T, Pietruck F, Franke GH (2006). The impact of living-related kidney transplantation on the donor's life. Transplantation.

[18] Tellioglu G, Berber I, Yatkin I (2008). Quality of life analysis of renal donors. Transplant Proc.

[19] Zargooshi J (2001). Quality of life of Iranian kidney donors. J Urol.

[20] Nejatisafa AA, Mortaz-Hedjri S, Malakoutian T (2008). Quality of life and life events of living unrelated kidney donors in Iran: a multicenter study. Transplantation.

[21] Smith M, Kappell D, Province M (1986). Living-related kidney donors: a multicenter study of donor education, socioeconomic adjustment, and rehabilitation. Am J Kidney Dis.

[22] Mjøen G, Stavem K, Westlie L, Midtvedt K, Fauchald P, Norby G, Holdaas H (2011). Quality of life in kidney donors. Am J Transplant.

[23] Clemens KK, Thiessen-Philbrook H, Parikh CR (2006). Psychosocial health of living kidney donors: a systematic review. Am J Transplant.

[24] Klop KW, Dols LF, Weimar W, Dooper IM, IJzermans JN, Kok NF (2013). Quality of life of elderly live kidney donors. Transplantation.

[25] Minnee RC, Bemelman WA, Polle SW (2008). Older living kidney donors: surgical outcome and quality of life. Transplantation.

[26] Sommerer C, Feuerstein D, Dikow R (2015). Psychosocial and physical outcome following kidney donation-a retrospective analysis. Transpl Int.

[27] Boter H, Mänty M, Hansen AM, Hortobágyi T, Avlund K (2014). Self-reported fatigue and physical function in late mid-life. J Rehabil Med.

